# Reliability of Change of Direction and Agility Assessments in Youth Soccer Players

**DOI:** 10.3390/sports8040051

**Published:** 2020-04-18

**Authors:** James H. Dugdale, Dajo Sanders, Angus M. Hunter

**Affiliations:** 1 Physiology Exercise and Nutrition Research Group, Faculty of Health Sciences and Sport, University of Stirling, Scotland FK9 4LJ, UK; a.m.hunter1@stir.ac.uk; 2 Department of Human Movement Science, Faculty of Health, Medicine and Life Sciences, Maastricht University, 6200 MD Maastricht, The Netherlands; dajosanders@gmail.com

**Keywords:** performance, adolescent, fitness testing, physical, maturation

## Abstract

Considering the vast physical and neural developments experienced throughout adolescence, the reliability of physical performance may vary in youth populations. This study aimed to examine the reliability of change of direction (COD) and agility tests in youth soccer players. Altogether, 86 youth soccer players, aged 13.6 ± 2.0 years, volunteered to participate. Data were collected from a modified 505 COD test (m505COD) and the Y-sprint drill in both pre-planned (Y-SprintPRE) and reactive (Y-SprintREACT) conditions during 2 sessions, 7 days apart. Anthropometric data including body mass, standing stature, and sitting height were also collected. COD and agility tests demonstrated good reliability (ICC = 0.81–0.91; CV = 1.2–2.0; *d* = 0.00–0.31; *p* < 0.01) for our entire sample. However, we observed a small negative relationship between age and intersession differences for the Y-SprintPRE (*r* = −0.28; *p* = 0.04), and moderate negative relationships between both age (*r* = −0.41; *p* < 0.01), and maturity offset (*r* = −0.39; *p* < 0.01) for the Y-SprintREACT. Although the COD and agility tests adopted within this study possess good intersession reliability, we observed greater intersession differences for younger and less mature individuals. We suggest that while COD and agility tests may provide meaningful objective data for monitoring the development of youth soccer players, these tests should be used with caution when evaluating younger, more immature athletes.

## 1. Introduction

The open and intermittent nature of competitive soccer places high demand on players to change mode or direction of movement on a constant basis [1,2,3]. During one season of English Premier League match play, between 1000 and 1500 discrete movement changes were observed per game, with changes in activity occurring, on average, every 3.5 s [4]. While change of direction (COD) sprints precede ~10% of goals in professional soccer matches, they are suggested to be of greater importance to defensive players attempting to pre-empt attacking players’ behaviour [5]. Consequently, the ability to meet these physical demands is crucial during soccer to enhance performance.

During soccer match play, changes in mode or direction of movement are largely due to external stimuli perceived by the player [6,7]. The skill of agility encompasses these demands and can be defined as “a rapid whole body movement with change of velocity or direction in response to a stimulus” [8]. However, also of importance during soccer match play is COD ability, defined as “the ability to change initial direction to a predetermined location and space on a field or court” [9]. Despite being misinterpreted in previous years, scientists have recently established agility and COD ability as independent skills [6,10,11], with little common variance [11,12,13,14]. Consequently, both qualities receive individual consideration when developing and assessing the physical qualities of team-sport athletes.

Nimphius and colleagues [10] suggest that a variety of tests can be administered when examining COD and agility performance—many of which vary in duration and physical demands. Approach velocity, acceleration and deceleration, and strength requirements can be challenged to varying degrees, dependent on the test selected [10,15,16]. When assessing COD performance, versions of the “505” test are most commonly selected due to their ability to challenge deceleration and reacceleration qualities, alongside providing comparative data between turn legs [10,17,18,19]. When assessing agility performance, however, versions of the Y-sprint drill are often implemented to maintain high sprint velocity and mimic the “cut” action, commonly observed during team and invasion sports [12,20,21,22]. Moreover, it is suggested that the presence of a stimulus may influence the physical demands elicited during a test [10,22,23,24]. Therefore, sufficient consideration should be given to both mechanical and conditional demands when selecting suitable COD and agility tests [15].

In addition to the importance of agility and COD abilities in professional soccer, Lloyd and Oliver (2012) suggest that these qualities should be prioritised within the training process during childhood and adolescence [25]. From a fundamental perspective, general motor skills and abilities related to COD and agility may be developed across childhood and adolescence [26]. However, considering the vast physical and neural developments experienced throughout maturation [27], the reliability of COD and agility performance may vary for this sample during objective testing [18,28]. Increases in stature, body mass, and sprint speed are evidenced across maturation [25,29,30,31], resulting in vast differences in performance during COD and agility tasks [30,32]. Moreover, it is generally accepted that maturity status and absolute strength are strongly associated, with strength performance and ability to absorb and elicit force improving throughout maturation [33]. The onset of “adolescence awkwardness” around peak height velocity is suggested to increase biological variance in physical test performances in adolescent athletes—notably, the ability to accelerate, decelerate and change direction in a consistent manner [18,30,34,35,36,37]. Performance of COD and agility actions also require complex technical competencies, which may not yet be fully developed in child and adolescent athletes [16]. For example, Dos’Santos and colleagues [38] observed improvements in COD performance and movement quality following a six-week training intervention centred around improving technical components of COD. Considering the high level of physical and technical demands elicited during COD and agility actions [16,39], greater variance in test performance may be observed in younger, more immature athletes, particularly during periods of rapid growth and development.

Few studies have examined the intersession reliability of COD test performance in youth athletes [18,28] and, to our knowledge, the intersession reliability of agility test performance in a broad age range of youth athletes is unknown. This information is important to coaches, so that they can differentiate between measurement noise and real change in COD and agility performance in their youth athletes. Accordingly, the purpose of this study was to examine the intersession reliability of COD and agility tests in youth soccer players. We hypothesise that the COD and agility tests will demonstrate good reliability but reliability will be lower within younger, less mature individuals as previously observed [18,28].

## 2. Methods

### 2.1. Participants

In total, 86 Scottish youth soccer players (mean ± SD (range)), aged 13.6 ± 2.0 (10.6–17.3) years, with a stature of 160.8 ± 13.7 (134.2–193.9) cm and mass of 50.1 ± 13.0 (27.5–78.7) kg, volunteered to participate in this study. Participants were signed to a junior-elite soccer academy playing at the top competitive level of the Scottish Football Association (SFA). Participants were categorised within the following age groups as specified by the SFA: U11/U12 (*n* = 31), U13/U14 (*n* = 23), and U15–U17 (*n* = 32). We obtained participant, parental/guardian and academy director consent and provided comprehensive written and oral explanations about the study prior to collecting any data. The study received institutional ethical approval (NICR 16/17 045-V2) in accordance with the Declaration of Helsinki.

### 2.2. Design

Participants completed 2 testing sessions, 7 days apart. During each session, we collected data from a modified 505 COD test (m505COD) and the Y-sprint drill in both pre-planned (Y-SprintPRE) and reactive (Y-SprintREACT) conditions. During testing session 1, we also collected anthropometric data including body mass, standing stature, and sitting height. Participants were familiar with the m505COD test from their regular fitness testing battery and were familiarised with the Y-SprintPRE and Y-SprintREACT by 1 pre-test. To account for circadian variability [40], both testing sessions were completed at the same time of day and during participants’ regular training times. Testing sessions were completed within a minimum of 48 hours following a competitive game, and in absence of strenuous exercise within 24 hours prior. Testing sessions were conducted indoors (~22 °C) on a synthetic 4G pitch. 

### 2.3. Procedures

Prior to conducting any tests, participants conducted a standardised warm up consisting of light aerobic activity, dynamic stretching, progressive sprinting, and submaximal pre-planned changes of direction. Following the standardised warm up, participants received verbal instruction and demonstrations from the research team immediately prior to conducting 2 familiarisation attempts to the left and right (4 attempts total) for each test. All participants completed the Y-SprintPRE trials prior to completing the Y-SprintREACT trials. Timing gates were adjusted to an appropriate hip height as per the mean stature of the sample group, and start positions were standardised as self-selected, staggered stance crouch starts from 0.7 m behind the start gate [41]. Data were collected using the Witty Dual Beam Timing System (Microgate, Bolzano, Italy) with the total time reported to the nearest 0.01 s. Recovery intervals between attempts were standardised at three minutes for each test.

### 2.4. Anthropometrics/Maturity Status

Standing stature and sitting height were assessed using a free-standing stadiometer (Seca, Birmingham, UK) and reported to the nearest 0.1 cm. Body mass was assessed using digital floor scales (Seca, Birmingham, UK) and reported to the nearest 0.1 kg. Somatic maturity estimates were made for all participants via non-invasive methods. A regression equation was then used to estimate years from age of peak height velocity (maturity offset) [42]. 

### 2.5. Change of Direction Tests

**Figure 1:**
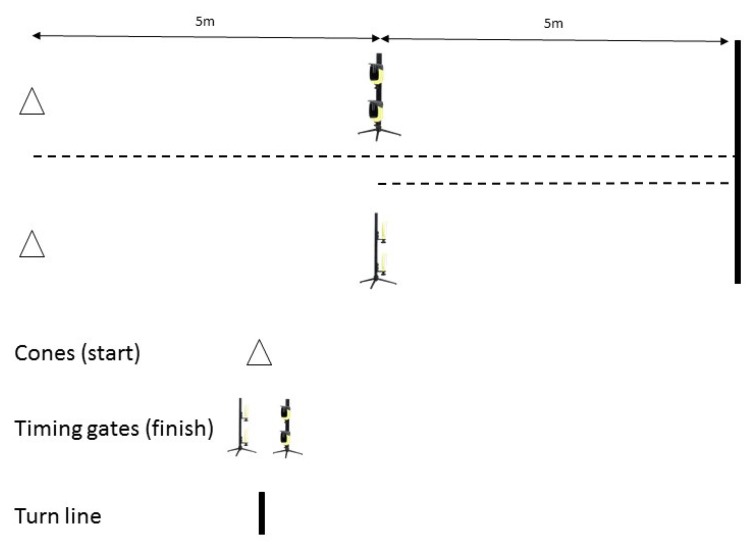
Set up for the m505COD test.

Pre-planned COD through the horizontal plane was assessed via the m505COD test [21] (Figure 1). The methodology for the m505COD was as per originally established methods [43], but shortening the linear sprint by 5 m in distance. Therefore, this involved a 10 m linear sprint from a static start, a 180° turn on a predetermined turn leg (right/left) ensuring contact with a designated line, and a 5 m return sprint through an identified finish line. The time taken to complete the final 5 m of the 10 m linear sprint, turn, and 5 m return sprint was recorded. 

**Figure 2:**
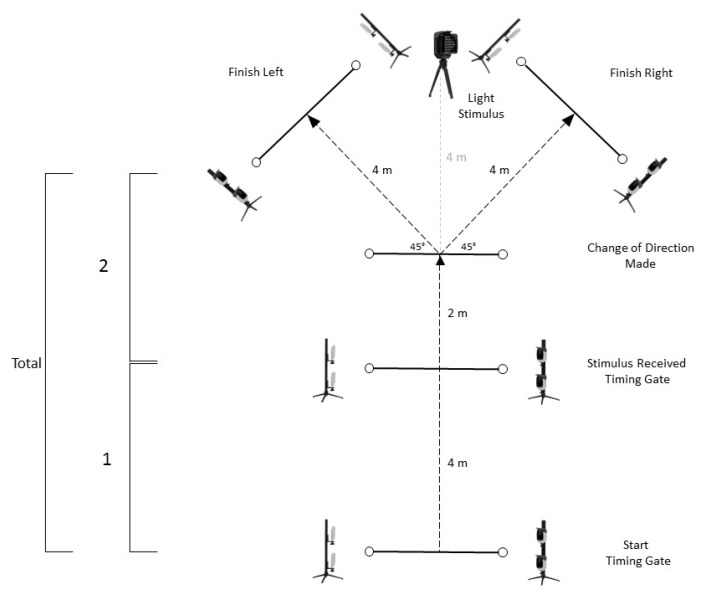
Set up of the Y-Sprint drill.

Pre-planned COD performance was also assessed using the Y-Sprint drill (Y-SprintPRE) (Figure 2). The Y-SprintPRE involved a 4 m linear sprint from a static start (phase 1), a further 2 m linear sprint followed by a 45 ° turn in a predetermined direction (right/left) and a 4 m sprint through an identified finish line (phase 2). For both the m505COD and Y-SprintPRE, participants completed two attempts at changing direction in each direction (right/left). Best attempts from each direction were selected, and a mean time from these two attempts was calculated and used for analysis [44].

### 2.6. Agility Test

Agility performance was assessed using a reactive version of the Y-Sprint drill (Y-SprintREACT). The protocol for the Y-SprintREACT included the addition of a Witty SEM light stimulus (Microgate, Bolzano, Italy), 10 m from the start position (Figure 2). Stimuli were displayed following a 0.5 s delay after crossing the “stimulus received” timing gate and received in a randomised order, with four trials completed for each participant. To account for performance variance during the Y-SprintREACT condition, the average of all four attempts was used for analysis [44,45]. Timings were provided for phase 1 (the initial 4 m linear sprint), phase 2 (the 2 m linear sprint followed by the change of direction), and total time (a combination of both phases) for both the Y-SprintPRE and Y-SprintREACT conditions.

### 2.7. Statistical Analysis

Prior to analysis, the assumption of normality was verified using the Shapiro–Wilk test. Descriptive data are reported as the mean ± standard deviation (SD). A two-way random effects intra-class correlation coefficient (ICC), with absolute agreement and reporting 95% confidence intervals (95%CI), was used alongside the coefficient of variation (CV) to evaluate relative test–retest reliability. Standardised effect size, reported as Cohen’s *d*, using the pooled SD as the denominator, was calculated to evaluate the magnitude of the test–retest differences. The standard error of measurement was calculated to provide an estimate of the precision of the tests and was calculated as SEM = *s* √1 – *r*, where *s* is the standard deviation of the measurements and *r* is the ICC [46]. The intra-session typical error of measurement (TE) was calculated and is expressed as a percentage. As per guidelines provided by Atkinson and Nevill (1998) [47] and Hopkins (2000) [48], the tests were deemed as reliable if they met the following criteria: good-to-excellent ICC (≥0.80), good CV (≤5%), and a trivial or small effect size (≤0.59). Relationships between variables were assessed using Pearson’s *r* correlation coefficient (±95%CI). Interpretation of the strength of the correlation coefficients are based on guidelines provided by Hopkins (2002) [49]: 0.00–0.09 trivial; 0.10–0.29 small; 0.30–0.49 moderate; 0.50–0.69 large; 0.70–0.89 very large; 0.90–0.99 nearly perfect; 1.00 perfect. Statistical significance was set at *p* < 0.05.

## 3. Results

The COD and agility tests selected within this study demonstrated good reliability (ICC = 0.81–0.91; CV = 1.2–2.0; *d* = 0.00–0.31; *p* < 0.01) for our entire sample. Notably, good intersession reliability was reported for the m505COD (ICC = 0.84–0.89; CV = 0.0–5.3), Y-SprintPRE (ICC = 0.87–0.91; CV = 0.0–5.1), and Y-SprintREACT (ICC = 0.81–0.86; CV = 0.3–4.6) for the age groups within our sample (Table 1). Intra-session TE for the COD and agility tests selected within this study was: m505COD: 2.0% (1.8; 2.2); Y-SprintPRE: 1.5% (1.3; 1.7); Y-SprintREACT: 4.3% (4.1; 4.5), for session 1 and session 2, respectively. 

There was a small negative relationship observed between age and intersession differences for Y-SprintPRE (*r* = −0.28; 95%CI: −0.07–−0.47; *p* = 0.04), and moderate negative relationships observed between both age (*r* = −0.41; 95%CI: −0.20–−0.60; *p* < 0.01), and maturity offset (*r* = −0.39; 95%CI: −0.20–−0.58; *p* < 0.01) for Y-SprintREACT (Table 2).

## 4. Discussion

Few studies have examined the intersession reliability of COD test performance in youth athletes [18,28] and, to our knowledge, the reliability of agility test performance in a broad age range of youth athletes is unknown. This study aimed to evaluate the intersession reliability of COD and agility tests across the entire spectrum of ages in a junior-elite soccer academy. The main finding of the present study was that COD and agility tests proved reliable for all age groups but small-to-moderate relationships between age/maturity offset and intersession performance differences were observed.

The COD tests examined within this study demonstrated a better intersession reliability than previously reported [18,28,50]. The better reliability demonstrated could potentially be explained due to the high performance standard of this sample, previously proposed as an influencer of test reliability in adolescent athletes [35,51]. Fundamental movement skills improve throughout childhood and adolescence through exposure to physical activity and sport [35,51]. Therefore, youth athletes from higher playing and performance standards may possess a higher relative training age [35,51] and, consequently, improved movement skills and physical competency. On the contrary, no influence on intersession fitness test reliability was observed across three distinct performance standards of Scottish youth soccer players [28], suggesting that performance standards may not influence the reliability of COD performance in this sample. We suggest that future empirical research further explore potential relationships between performance standard and intersession test reliability in samples of youth athletes across childhood and adolescence.

Considering the varied physical demands imposed by different COD tests [10,15,16], the shorter test duration and singular acute change of direction may explain the improved intersession reliability we observed for the Y-SprintPRE compared to previous research [28,50]. The ability to change direction is angle dependent and affected by entry velocity into the COD [10,15,16]. Consequently, the technique and loading required during execution of a 45° cut during forward sprinting (as required by the Y-SprintPRE) compared to a 90°–180° COD (as required by tests included in comparable research) [15,28,50] will vary. We suggest that the Y-SprintPRE places a greater emphasis on linear sprint ability, potentially reducing the challenge placed upon the typically underdeveloped eccentric strength qualities and limited movement competency observed in youth athletes [18,30,34,36,37]. On the contrary, despite selecting similar tests and examining a comparable sample, we demonstrated substantially better intersession reliability for the m505COD test than Taylor et al. [18]. While Taylor and colleagues [18] selected a modified 505 test as per the “English Premier League EPPP” curriculum, we selected the m505COD previously established by Gabbett et al. [21]. Methodological differences in approach distance and start distance behind the gates are present between the m505COD and EPPP modified 505 test, potentially explaining these differences in observed reliability. Moreover, in the present study, we employed a dual-beam timing system to capture our data, as required to derive accurate and reliable timings over short sprint distances [41,52]. Considering the magnitude of difference in intersession reliability observed between the present study and Taylor et al. [18], we suggest that further investigation into the intersession reliability of modified 505 tests, within youth athlete populations, is warranted.

Comparative to COD assessment, relatively little is known regarding the intersession reliability of agility tests in samples of youth athletes. We found that the Y-SprintREACT test (our assessment of agility performance) demonstrated good intersession test reliability in our sample of youth soccer players. Despite being classified as independent skills [6,10,11], the physical demands of both COD and agility performance are similar, challenging linear sprint, acceleration, and deceleration capacities [13,45]. When compared to human stimuli, reacting to generic light stimuli requires manipulation to body position, subsequently increasing the external loading on the knee when performing the cutting movement [24]. This form of stimulus removes the ability of performers to interpret subtle manipulations to body position and early pattern recognition, prevalent during sport-specific scenarios, and subsequently placing greater demand on physical characteristics during agility task completion [13]. Therefore, the implementation of a generic light stimulus during the Y-SprintREACT drill, in our study, may have imposed greater physical demands compared to tests, including a more ecologically valid stimulus. The use of commercially available light timing gate systems within an applied setting is, however, extremely common and, therefore, knowledge provided by the present study may help better inform coaches and practitioners when using this equipment. 

While intersession reliability was acceptable across all age groups, small-to-moderate negative relationships were observed between age/maturity offset and between trial performance differences for both Y-SprintPRE and Y-SprintREACT tests. Although these were small-to-moderate relationships, this finding suggests that poor motor coordination, limited physical literacy, and underdeveloped strength capacities associated with less mature and chronologically younger athletes, evident within our sample, may result in greater variability in test performance [30,35,51]. To combat these issues, we suggest that COD ability, along with its contributing physical qualities, be implemented in training programmes during childhood and early adolescence to ensure learning and development of these movements and skills as early as possible during athletic development [25,26,30]. These relationships were not, however, observed for the m505COD test. Considering that the physical and technical demands of the m505COD test are greater than those elicited by the Y-Sprint drill, this finding is surprising. It has been suggested that training exposure may offset the influence of the inconsistency of test performance in youth athletes. Moreover, adolescent athletes have been shown to pace their run up when performing the traditional 505 COD test because of the increased physical demand of a fast entry velocity [53]. We suggest that technical approaches to this test and learning effects associated with regular test completion may provide some explanation for this finding, and that prior exposure to the test should be noted when considering the intersession reliability of COD tests.

This study is not without its limitations. As discussed, measures of COD and agility possess differing physical, mechanical and conditional demands [10,15]. Similarly, the technical competency of COD movements may affect consistency in performance [15,38]. We acknowledge that our findings may not be transferrable to other COD and agility tests, or within other sample populations, and encourage further research in this area. Finally, we employed a generic light stimulus for our test of agility performance. Given the physical and conditional differences between light and human stimuli [10,24], we propose that the reliability of agility performance using a human stimuli needs to be established independently, and encourage using results from our study only when selecting a generic light stimuli as per our methods. 

## 5. Conclusions

Our findings suggest that the COD and agility tests adopted within this study possess good intersession reliability, and therefore provide meaningful and objective data for monitoring the development of youth soccer players. The better reliability demonstrated in the present study, comparative to previous research in a comparable sample, may provide rationale for selecting these tests opposed to others when evaluating COD and agility performance in youth soccer players. However, due to the tendency for greater between-trial differences in younger and less mature individuals, the requirement and suitability of COD and agility tests for the evaluation of these populations could be considered. We suggest that substantial amounts of measurement noise, in these tests, may prohibit coaches from being able to identify real change in COD and agility performance when assessing youth athletes.

## Figures and Tables

**Table 1:** Intersession test–retest reliability for COD and agility tests displayed by age bracket.

			U11/U12	U13/U14	U15–U17
			(n = 31)	(n = 23)	(n = 32)
Change of Direction	m505COD	Session 1 (x ± SD) (s)	2.61 ± 0.14	2.43 ± 0.06	2.33 ± 0.08
		Session 2 (x ± SD) (s)	2.60 ± 0.14	2.42 ± 0.11	2.34 ± 0.11
		ICC (95%CI)	0.88 (0.82–0.92)	0.84 (0.65–0.93)	0.89 (0.78–0.95)
		CV (%)	1.8	1.6	1.6
		SEM (95%CI) (s)	0.05 (0.10)	0.04 (0.07)	0.03 (0.07)
		*d*	0.07	0.11	0.10
	Y-SprintPRE	Session 1 (x ± SD) (s)	1.86 ± 0.13	1.84 ± 0.07	1.75 ± 0.07
		Session 2 (x ± SD) (s)	1.86 ± 0.12	1.83 ± 0.07	1.77 ± 0.06
		ICC (95%CI)	0.91 (0.87–0.94)	0.87 (0.72–0.94)	0.91 (0.82–0.95)
		CV (%)	1.5	1.2	1.4
		SEM (95%CI) (s)	0.04 (0.07)	0.03 (0.05)	0.02 (0.04)
		*d*	0.08	0.13	0.31
Agility	Y-SprintREACT	Session 1 (x ± SD) (s)	2.40 ± 0.18	2.28 ± 0.12	2.35 ± 0.14
		Session 2 (x ± SD) (s)	2.40 ± 0.18	2.29 ± 0.11	2.35 ± 0.15
		ICC (95%CI)	0.82 (0.59–0.92)	0.81 (0.55–0.92)	0.86 (0.71–0.93)
		CV (%)	2.0	1.7	1.7
		SEM (95%CI) (s)	0.08 (0.15)	0.05 (0.10)	0.05 (0.11)
		*d*	0.00	0.00	0.07

n = sample size; x ± SD = mean ± standard deviation; ICC = intra-class correlation, all *p* < 0.01; 95%CI = 95% confidence interval; CV = coefficient of variation; SEM = standard error of measurement; *d* = Cohen’s *d* effect size.

**Table 2:** Relationships between age/maturity offset and intersession differences in performance for COD and agility tests.

	m505COD	Y-SprintPRE (Total)	Y-SprintREACT (Total)
Age (95%CI)	−0.14 (−0.10 – −0.36)	−0.28 * (−0.07 – −0.47)	−0.41 ** (−0.20 – −0.60)
Maturity Offset (95%CI)	−0.19 (−0.03 – −0.39)	−0.19 (−0.01 – −0.38)	−0.39 ** (−0.18 – −0.57)

* *p* < 0.05; ** *p* < 0.01; 95%CI = 95% confidence interval.
